# Pre-operative intravitreal bevacizumab for tractional retinal detachment secondary to proliferative diabetic retinopathy: the Alvaro Rodriguez lecture 2023

**DOI:** 10.1186/s40942-023-00467-8

**Published:** 2023-04-18

**Authors:** J. Fernando Arevalo, Bradley Beatson

**Affiliations:** Wilmer Eye Institute, Johns Hopkins School of Medicine, 600 N Wolfe St; Maumenee 713, Baltimore, MD 21287 USA

**Keywords:** Tractional retinal detachment, Proliferative diabetic retinopathy, Bevacizumab, anti-VEGF

## Abstract

The treatment of proliferative diabetic retinopathy (PDR) has evolved significantly since the initial use of panretinal photocoagulation as a treatment in the 1950s. Vascular endothelial growth factor inhibitors have provided an effective alternative without the risk of peripheral vision loss. Despite this, the risk of complications requiring surgical intervention in PDR remains high. Intravitreal bevacizumab has shown promise as a preoperative adjuvant to vitrectomy for PDR complications, albeit with a purported risk for tractional retinal detachment (TRD) progression in eyes with significant fibrous proliferation. Here we will discuss anti-VEGF agent use in PDR and its role in surgical intervention for PDR complications including TRD.

## Background

Diabetic retinopathy (DR) is a common microvascular complication of diabetes mellitus that can progress to vision-threatening complications and is a leading cause of blindness amongst working-age adults in the United States [1]. It has an estimated prevalence of 35–40% and 85% amongst individuals with type 2 diabetes and type 1 diabetes respectively, and the number of Americans with DR is projected to reach 16 million individuals by 2050 [2–4]. Proliferative diabetic retinopathy (PDR) is a vision-threatening progression of DR with a prevalence of 7% amongst adults with diabetes [5, 6]. PDR is defined by neovascularization and fibrovascular proliferation at the vitreoretinal interface, which can result in tractional forces on the retina and subsequent complications such as tractional retinal detachments (TRDs) and vitreous hemorrhages. This fibrovascular proliferation is believed to be due to upregulation of several angiogenic factors in eyes affected by PDR with associated retinal ischemia, with vascular endothelial growth factor (VEGF) in particular having been found to have a prominent role [7–9].

The use of bevacizumab (Avastin®, Genentech Inc., San Francisco, CA), an anti-VEGF agent, to treat neovascularization in PDR began to popularize following its reported success in treating neovascular age-related macular degeneration [10–12]. In 2006, a subsequent case series by Avery et al. demonstrated its potential applicability to PDR [13]. In the following years, use of bevacizumab was reported as a preoperative adjunct therapy for repair of TRD in the setting of PDR [14–17]. This was hypothesized to reduce abnormal vasculature reactivity prior to pars plana vitrectomy (PPV) due to its angiogenic properties, thus potentially allowing for lower rates of intraoperative hemorrhage and iatrogenic retinal tears. Here we will provide a broad overview of the treatment of PDR with anti-VEGF agents and PPV, with a particular focus on the use of bevacizumab as an adjunct to treatment of TRD in the setting of PDR.

## History of anti-VEGF agent use in PDR

Before the discovery of VEGF’s role in PDR and the use of intravitreal anti-VEGF agents for PDR, standard of care for these patients involved laser photocoagulation treatment since its first reported use in 1959 [18]. In 1981, the Diabetic Retinopathy Study Research Group reported the use of photocoagulation reduced the risk of severe vision loss by at least 50% in patients with PDR [18]. While laser photocoagulation was widely accepted as an effective treatment since that time, the principal mechanism by which PDR caused neovascularization and the underlying biochemical effect of photocoagulation was not well understood. It was not until 1994 when it was discovered that VEGF increased in the vitreous in response to retinal hypoxia in eyes with PDR [7, 8, 19]. Along with that finding, Aiello et al. reported that levels of VEGF were decreased in PDR eyes following laser photocoagulation relative to eyes without photocoagulation treatment [8]. This discovery led to the current understanding that by reducing the amount of ischemic peripheral retinal tissue via photocoagulation, total VEGF production and associated neovascularization is stunted. This work was followed by nonhuman primate studies showing intravitreal VEGF inhibition prevented neovascularization in response to induced retinal ischemia, paving the way for future clinical studies using anti-VEGF agents in humans [20].

Following these discoveries, several intravitreal anti-VEGF agents underwent clinical trials for use in neovascular ocular diseases, culminating with the 2006 approval of ranibizumab (Lucentis, Genentech, South San Francisco, California, USA) for neovascular age-related macular degeneration [21]. Other notable drugs approved for neovascular eye diseases included pegaptanib sodium (Macugen; OSI Eyetech Pharmaceuticals, Melville, NY, USA), and aflibercept (Eylea, Regeneron Pharmaceuticals, Inc., Tarrytown, NY) [22, 23]. Bevacizumab, an anti-VEGF agent approved for metastatic colon cancer, also showed efficacy in treatment of PDR-related neovascularization when injected intravitreally as an off-label use requiring the use of compounding pharmacies [13, 24]. The relative effectiveness of these drugs were long considered comparable for the use of PDR and choice was often determined by cost or patient-specific factors. There is a notable lack of large, prospective studies examining relative efficacy of these drugs for treatment of PDR. In 2015, the Diabetic Retinopathy Clinical Research Network (DRCR) released a report comparing the relative efficacy of intravitreal bevacizumab (IVB), intravitreal aflibercept (IVA), and intravitreal ranibizumab (IVR) for diabetic macular edema (DME) [25]. DRCR reported comparable efficacy amongst all drugs for treatment of DME when baseline visual acuity 20/40 or better. For patients with baseline visual acuity worse than 20/40, the IVA group obtained significantly better visual acuity scores than either IVB or IVR. However, one year later, the DRCR released a report highlighting the relative cost-effectiveness of IVA, IVB, and IVR during their study. This report noted that the prices of IVA and IVR would need to decrease by 69% and 80% respectively to maintain the same cost effectiveness of IVB [26]. Consequentially, while many anti-VEGF agents surfaced for treatment of neovascular ophthalmic diseases, off-label use of IVB proved to be a cost-effective and popular option.

One cross-sectional report found that from 2012 to 2019, the use of anti-VEGF agents began to increase significantly in the United States while panretinal photocoagulation (PRP) rates decreased [27]. This shift was likely spurred by the publishing of Protocol S by the DRCR in 2015, which found IVR to be non-inferior to PRP for treatment of PDR [28]. In 2018, the DRCR released the 5-year results of this study, which found similar visual acuity results, although the IVR group experienced less visual field loss and a lower rate of diabetic macular edema. Protocol S was followed by the CLARITY phase 2b clinical trial in 2017, which found that IVA provided a significantly better visual acuity difference than PRP at the end of the 1-year follow up [29]. However, it is important to note that anti-VEGF treatment is reliant on patient compliance, as the effect is transient and dependent upon repeated injections. The large number of patients lost to follow-up in Protocol S emphasized this risk [28].

## Surgical intervention for late complications in PDR

While PDR can be managed with PRP or IVB in the majority of cases, in some eyes ongoing tractional forces due to fibrovascular membrane contraction at the vitreoretinal interface can result in complications such as persistent/recurrent vitreous hemorrhage or TRD. These complications can complicate the clinical course and require surgical intervention.

Historically, PPV was only performed in eyes with severe vitreous hemorrhage lasting at least one year or for TRD involving the macula. In 1985, the Diabetic Retinopathy Vitrectomy Study Research Group (DRVS) studied the use of early vitrectomy in eyes with visual acuity reduced to 5/200 or less for at least one month due to severe vitreous hemorrhage or sooner if a macula-involving TRD was present [30]. The control (deferral) group underwent PPV after 1 year of severe vitreous hemorrhage, or sooner if macula-involving TRD was present. The study found that at the 2-year follow-up visit, 24.5% of eyes in the early vitrectomy group had a visual acuity of 10/20 or better compared to 15.2% in the deferral group (p = 0.01). These benefits persisted at the 4-year follow-up visit [31]. These findings established the current role of earlier surgical intervention in the clinical course of PDR complications.

Complications requiring surgical intervention remain a moderately frequent occurrence in the treatment of PDR, even in the era of anti-VEGF treatment. The 5-year results of Protocol S showed 21 eyes (15%) in the IVR group underwent PPV, while 39 eyes (22%) of the PRP group underwent PPV (p = 0.008) [32]. Retinal detachment (RD) was a frequent cause of vitrectomy in the study, with 12 eyes (7%) in the IVR group experiencing RD, and 30 eyes (18%) in the PRP groups experiencing RD (p = 0.004) [32]. The CLARITY trial reported a lower number of eyes requiring PPV following non-surgical treatment, with 1 eye (1%) in the IVA group and 7 eyes (6%) in the PRP group requiring PPV, although the difference between the groups was not significant (p = 0.066) [29]. It is also worth noting that the follow-up time for the CLARITY trial was limited to one year. In 2021, the India Retinal Disease Study Group reported that 326 eyes (31.4%) of their 1038 eye cohort with PDR required PPV, further emphasizing the role of follow-up duration and monitoring these patients closely for development of complications over extended periods of time [33].

Visual and anatomic outcomes of PPV for late complications of PDR have improved significantly over time. In 1983, Rice et al. reported a final anatomic success rate of 66% for repair of TRD due to PDR using PPV in 197 eyes [34]. Visual outcomes did not fare significantly better, with only 57% of patients recording an improved visual acuity compared to presentation and 35% recording a worse visual acuity at end of follow-up. Lens removal and iatrogenic retinal breaks were cited as the two factors associate with a poorer visual prognosis (p < 0.002 and p < 0.01, respectively) [34]. In 2018, Storey et al. investigated visual and anatomic outcomes of PPV for TRD due to PDR in 403 eyes [35]. This study reported a single surgery anatomic success rate of 87.6% and a final anatomic success rate of 92.6% at final follow-up after 6 years. 57.6% of eyes recorded an improved visual acuity of at least two lines by final follow-up, while 19.9% recorded a worsened visual acuity of at least two lines [35]. However, despite recent advancements, outcomes remain suboptimal relative to uncomplicated RD repair. The final anatomic success rate recorded by Storey et al. is lower than that typically seen in recent large studies of uncomplicated rhegmatogenous RD, where final anatomic success rate approaches 100% [35–37].

Several factors may lead to poor outcomes in these eyes. Extensive fibrovascular proliferation can limit dissection, and intraoperative bleeding can limit visualization during PPV and lead to iatrogenic retinal breaks, thus increasing risk for further complications and subsequent redetachment. Even in optimal cases with few negative prognostic factors, visual outcomes may be unpredictable [38].

## IVB as an adjunct for TRD in PDR

The poor outcomes associated with PPV for PDR complications generated interest in optimizing surgical conditions and reducing intraoperative complications. In 2006, Chen and Park published a case report on the use of IVB as a preoperative adjunct for repair of TRD associated with PDR in a 27-year-old male [14]. Chen and Park theorized that preoperative IVB would reduce abnormal vasculature prior to PPV and decrease the likelihood of significant intraoperative hemorrhage. One week after administering 1.25 mg IVB and immediately prior to surgery, they found that there was a significant reduction in neovascularization and minimal intraoperative bleeding [14].

**Fig. 1:**
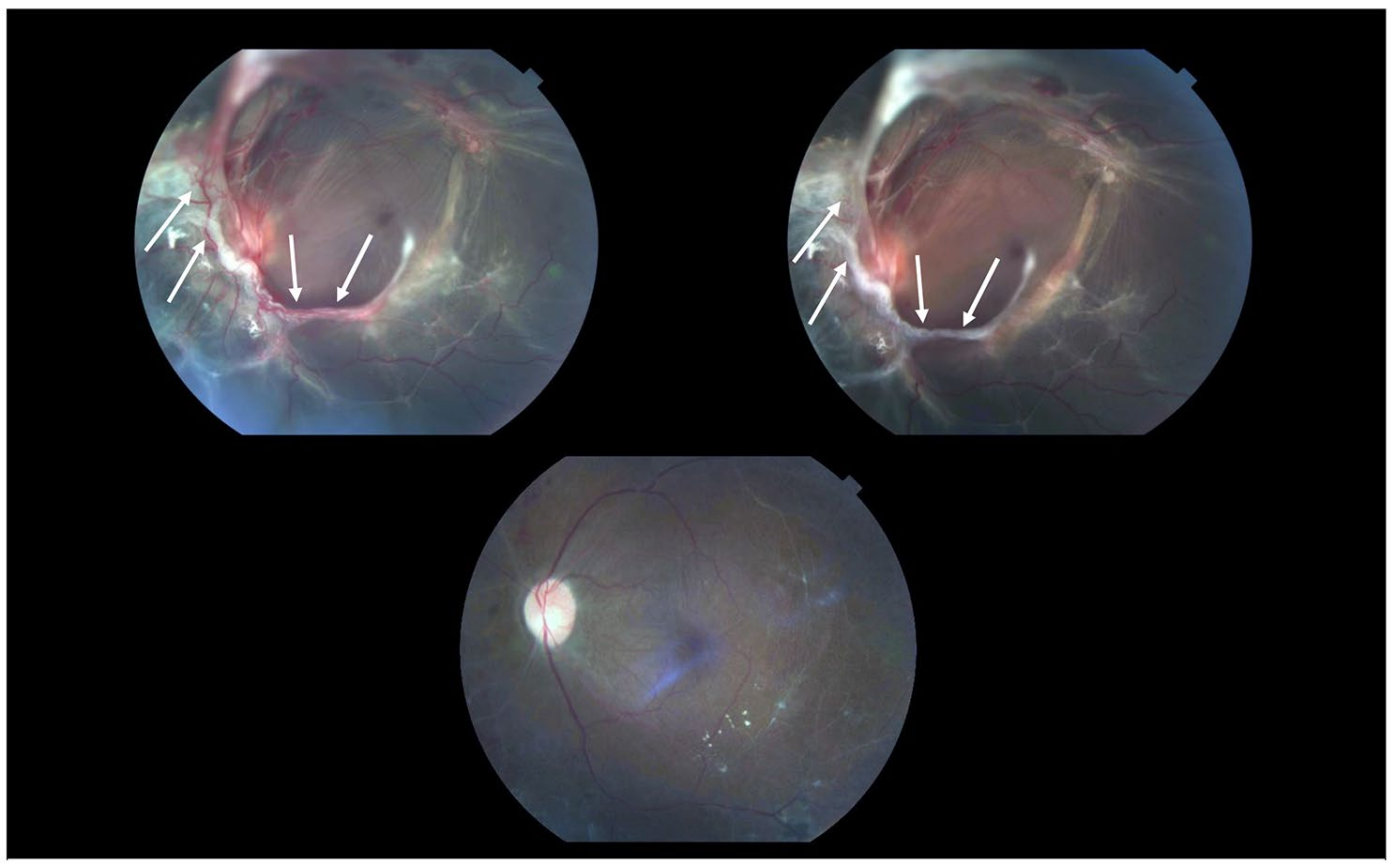
This is a case of a 46-year-old male who presented with PDR and a best-corrected visual acuity of 20/400 Top left: Significant fibrovascular membranes at time of presentation clearly visible and marked by white arrows. Top right: 3 days following preoperative IVB and one day before PPV, patient showed significant reduction in vascular proliferation. Bottom middle: 12 months following PPV with C_3_F_8_ tamponade and reattachment of the retina, patient had a best-corrected visual acuity of 20/70 Abbreviations: PDR-proliferative diabetic retinopathy; IVB-intravitreal bevacizumab; PPV-pars plana vitrectomy; C3F8-perfluoropropane gas.

Figure 1 demonstrates a case which shows a significant reduction in vascular proliferation 4 days following IVB administration preoperatively. Preoperative IVA has also shown promise in one randomized clinical trial as a more effective alternative to IVB, although there has been minimal follow-up data since this report in 2019 [39].

Following the initial report of preoperative IVB use, Rizzo et al. compared the use of PPV with preoperative IVB to PPV alone in 22 eyes with severe PDR [40]. They found that preoperative IVB resulted in significant decreases in intraoperative time, intraoperative bleeding, iatrogenic retinal tears, and use of endodiathermy. A meta-analysis performed by Zhao et al. supported these results [41]. Yeoh et al. presented similar findings in their case series of 18 eyes, reporting that IVB resulted in increased ease of surgery in complex eyes with TRD associated with active neovascularization [42]. The IBeTra study later quantified the difference in intraoperative bleeding during PPV for TRD and found that the mean erythrocyte count in the vitrectomy cassette fluid was 14 865 × 10^3^ in the IVB before PPV group and 176 240 × 10^3^ in the PPV only group (p < 0.0001) [43]. Adjunctive IVB has also been found to reduce the incidence of recurrent postoperative vitreous hemorrhage in the first 4 weeks following PPV, potentially reducing the need for subsequent PPV and improving visual outcomes [15, 44–47].

## Development or progression of TRD following preoperative IVB

Despite these positive results, several case studies reported development or progression of TRD following preoperative IVB administration [48–50]. This phenomenon was first identified in 2008, when Arevalo et al. reported a TRD development or progression rate of 5.2% shortly following preoperative IVB administration in 11 eyes [48]. While some reports found that the neovascular proliferation was reduced following IVB administration, there was an increase in fibrous tissue proliferation. It has thus been hypothesized that unintended contraction of the fibrovascular membrane and elevation of the retina may be the underlying cause of TRD progression [48–50]. This hypothesis was supported in 2012 when Geest et al. demonstrated that connective tissue growth factor (CTGF) in the vitreous increased in response to IVB and CTGF correlated positively with the level of fibrosis in PDR patients [51].

The rate of TRD incidence or progression following IVB administration has varied wildly, ranging from 1.5 to 18% in larger studies [16, 48, 52, 53]. Furthermore, the risk of TRD following IVB has not been found to be distributed equally across the patient population. In 2009, Oshima et al. found that absence of prior PRP and the presence of a ring-shaped fibrovascular membrane increased risk for IVB-induced complications [16]. In the same year, Ishikawa et al. noted that performing PPV within 5 days following IVB administration decreased the risk for extensive fibrosis and associated surgical complications [54]. Age and duration of diabetes history were also found to be factors that increased risk for TRD and poor visual outcomes following preoperative IVB [17].

In 2011, the Pan American Collaborative Retina Study Group (PACORES) published a retrospective study examining risk factors for TRD development following preoperative IVB in 698 patients [53]. The study found that more than 15 years of diabetes history, an IVB dose of 2.5 g or more, and performing PPV more than 13 days following IVB administration increased the risk for TRD development or progression. Since this study was retrospective without a control group, it was not possible to rule out TRD as a natural sequela to severe PDR. Consequentially, it was followed up in 2019, where PACORES published a prospective study of 224 eyes that reported reduced intraoperative bleeding, improved surgical field visualization, and reduced intraoperative and postoperative complications in the IVB + PPV group compared to the control (sham + PPV) group [55]. The rate of TRD progression was 2.9%, although visual acuity was improved after PPV in all cases of TRD progression, leading the authors to suggest preoperative IVB was safe and effective, despite the risk for TRD [55].

While several reports suggested increased risk of TRD progression and incidence following preoperative IVB, Bressler et al. published a pooled analysis of PDR eyes in five DRCR protocols that found no increased risk for TRD following IVB administration relative to the control groups (laser photocoagulation, sham, or intravitreal saline) [56]. It is worth noting that this study did not include eyes for which prompt vitrectomy was already planned, as the exclusion criteria included eyes with pre-existing TRD.

## Conclusions

The advent of anti-VEGF agents has changed the landscape of PDR treatment significantly, providing a non-inferior or even superior alternative to PRP in some patients. However, PDR remains a disease with a high risk for complications and subsequent surgical intervention. Intravitreal IVB has proven an effective adjuvant when given preoperatively for PPV due to PDR complications, where it is able to reduce intraoperative bleeding, reduce iatrogenic retinal tears, shorten operative time, and decrease postoperative vitreous hemorrhage. There does exist a risk for fibrous proliferation following preoperative IVB use, potentially causing progression or development of TRD. Current literature suggests this risk can be decreased by judicious use of preoperative IVB in patients with a long history of diabetes, using a lower dose of IVB (1.25 mg or lower), and by decreasing the time from IVB to PPV. Further research to compare the risk IVB has in eyes that require PPV and those that do not require PPV would be useful.

## Data Availability

Not applicable.

## List of Abbreviations

DR: diabetic retinopathy
PDR: proliferative diabetic retinopathy
PPV: pars plana vitrectomy
VEGF: vascular endothelial growth factor
PRP: panretinal photocoagulation
RD: retinal detachment
TRD: tractional retinal detachment
IVB: intravitreal bevacizumab.
